# Medical Items

**Published:** 1897-12

**Authors:** 


### MEDICAL ITEMS.

Doctors are under obligations to the Antikamnia Chemical Company for a neat and acceptable pocket case containing tablets of the Antikamnia combinations.

PRESIDENT McKINLEY appointed Dr. Newton L. Bates Surgeon-General of the Navy to succeed Dr. J. Rufus Tryon. Dr. Bates died within two weeks after his appointment, and the place passed to Medical Director W. K. Van Reypen.

AN Irish brakeman in the railroad yards was hurt by the train and his friends offered to send for a physician. They asked: "Do you want an allopath or homeopath?" He replied: "It don't matter—all paths lead to the grave."—*Ex.*

THE plague in Bombay is rapidly assuming its former proportions. During the last week of September there were sixty deaths from that disease in the city of Bombay, and many more in the presidency. The plague has reappeared in Kurrachee and is spreading to other towns, having already invaded Sholapur.—*Ex.*

A sensational story has been started by a New York daily to the effect that Henry M. Stanley is gradually turning black—becoming a negro in fact! That his skin is already a mulatto-colored and taking a deeper hue daily. It is added that "this remarkable occurrence is attributable to the transfusing of African (negro) blood into his veins on several occasions, while in the wilds, for the purpose of immunizing him against the dense miasm so prevalent in the low lands of the Dark Continent.—*Medical Times.*

IF albuminuria complicates pregnancy (Parvin in *Dunglinson's Coll. and Clin. Rec.*) to a serious extent, the patient should be placed upon an almost absolute milk diet, with lime-water to aid in its digestion, if necessary. Hot baths should be frequently taken and the bowels should be kept freely open. This complication should be thought of and examinations made for its determination in every case which comes under the physician's care in time to admit of it.—*Buffalo Med. Jour.*

---

THE origin and course of the present epidemic of yellow fever proves the utter untrustworthiness and inadequacy of local quarantine. Nearly all previous epidemics have taught the same lesson. A uniform law, with adequate authority to sustain its impartial and fearless enforcement, is the only defense against these perennial invasions. Some department of the national government is the proper agent to enforce such a law. Uniformity and authority can be secured in no other way. The first duty of this branch of the government, however instituted, should be the establishment of a national quarantine worthy the name.—*Medical News.*

---

PROF. BROUARDEL, in a recent lecture, related the following case: A man had a pharyngeal abscess, so deeply seated that his medical attendant was afraid to meddle with it. One night a burglar broke into the house, and on the sick man's calling for help tried to throttle him. The abscess burst, deluging the burglar with pus, and causing him to beat a rapid retreat. His intended victim experienced intense relief, and made a rapid recovery.—*Ex.*

Whether Professor B. recommended burglars for pharyngeal abscesses, or pharyngeal abscesses for burglars, is not stated.

DR. W. H. JOHNSON, dean of the Birmingham Medical College, writes to the *Journal* that the offer of $10 a head for medical students, payable to any doctor who would corral, ship and deliver to the college any embryo doctor in good condition, was made without the knowledge, consent or approval of the faculty of that school, and he adds: "We positively decline to abide by any such proposition and hereby retract it. Our school will stand on its merits or not at all." He requests that all medical journals which made a note of the startling innovation in methods of securing students shall do the school justice by printing his explanation.—*American Journal of Medicine and Surgery.*

---

IN January, 1898, the Philadelphia Medical Publishing Company will begin the publication of a weekly medical journal, to be called *The Philadelphia Medical Journal.* The management of the company will be intrusted to a board of trustees on which will be representatives of leading medical schools. The journal will be conducted solely in the interest of medical science and the medical profession, and it will be free from undue influence of individuals, firms and schools. The editorial management will be entrusted to Dr. George M. Gould, whose high reputation as a medical editor guarantees a vigorous, high-toned and entertaining publication.

This new journal seems to be starting out on the right lines, and if it continues to hew to this mark it will deserve and achieve success.

---

A WASHINGTON dispatch to the *Tribune*, dated November 15, 1897, stated that Surgeon-General Sternberg, of the Army; Dr. Horlbeck, of Charleston; Dr. Josiah Hartzell, of Canton, Ohio; Dr. Samuel H. Durgan, of Boston; Dr. A. H. Doty, of New York, and Dr. S. R. Olliphant, of New Orleans, the president of the Louisiana State Board of Health, all members of the American

Public Health Association, called at the White House to-day. They saw the President and urged him to incorporate in his message a recommendation that a commission be appointed to go to Havana to study the subject of yellow fever and the manner in which it is brought to the United States. They declared that good regulations in Havana would do more to prevent yellow fever in the United States than the best quarantine regulations that could be adopted and enforced. The President said he would give their suggestions due consideration.—*Buffalo Med. Journal.*

---

HARRISON ALLEN, M.D., professor of comparative anatomy in the medical school of the University of Pennsylvania, died suddenly November 14th of what is believed to have been heart disease. Dr. Allen was born in Philadelphia in 1841. He graduated from the University of Pennsylvania in 1861 and soon after entered the regular army. He was stationed at Washington, and in 1865, Dr. Allen, then only twenty-four years old, was called to the chair of comparative anatomy and zoology, which he held until May, 1895. He was the author of many papers and books on many phases of medicine. At the Columbian Exposition Dr. Allen was one of the judges on anthropology. He was a member of the Academy of Natural Sciences, Natural History Society of Boston, Pathological Society of Philadelphia, Biological Society of Washington, Philadelphia County Medical Society, Historical Society of Texas and the American Association of Anatomy. He was the correspondent of the Natural Sciences Society of Chili, and was corresponding secretary of the Academy of Natural Sciences in 1868, vice-president of the Pathological Society in 1877, president of the American Laryngological Association in 1866, president of the American Association of Anatomy from 1891 to 1893. He visited Europe in 1878 and 1890 as delegate to the International Medical Congress at Berlin.—*Medical Review.*

### WHEN DOCTORS DISAGREE.

He looked at my tongue and he shook his head—
This was Doctor Smart—
He thumped on my chest, and then he said:
"Ah! there it is! Your heart!
You mustn't run—you mustn't hurry!
You mustn't work—you mustn't worry!
Just sit down and take it cool;
You may live for years, I cannot say;
But, in the meantime, make it a rule
To take this medicine twice a day!"

He looked at my tongue and he shook his head—
This was Doctor Wise—
"Your liver's a total wreck," he said,
"You must take more exercise!
You mustn't eat sweets,
You mustn't eat meats,
You must walk and leap, you must also run;
You mustn't sit down in the dull old way;
Get out with the boys and have some fun—
And take three doses of this a day!"

He looked at my tongue and he shook his head—
This was Doctor Bright—
"I'm afraid your lungs are gone," he said,
"And your kidney isn't right.
A change of scene is what you need,
Your case is desperate, indeed,
And bread is a thing you mustn't eat—
Too much starch—but, by the way,
You must henceforth live on only meat—
And take six doses of this a day!"

Perhaps they were right, and perhaps they knew,
It isn't for me to say;
Mayhap I erred when I madly threw
Their bitter stuff away;
But I'm living yet, and I'm on my feet,
And grass isn't all that I dare to eat,
And I walk and I run, and I worry, too,
But, to save my life, I cannot see
What some of the able doctors would do
If there were no fools like you and me.

—S. E. KISER, in *Cleveland Leader.*

